# High strength metallic wood from nanostructured nickel inverse opal materials

**DOI:** 10.1038/s41598-018-36901-3

**Published:** 2019-01-24

**Authors:** James H. Pikul, Sezer Özerinç, Burigede Liu, Runyu Zhang, Paul V. Braun, Vikram S. Deshpande, William P. King

**Affiliations:** 1 Department of Mechanical Engineering and Applied Mechanics, University of Pennsylvania, Philadelphia, Pennsylvania USA; 2 Department of Mechanical Engineering, Middle East Technical University, Ankara, Turkey; 3 Department of Engineering, University of Cambridge, Cambridge, UK; 4 Department of Materials Science and Engineering, University of Illinois at Urbana-Champaign, Urbana, Illinois USA; 5 Department of Mechanical Science and Engineering, University of Illinois at Urbana-Champaign, Urbana, Illinois USA

## Abstract

This paper describes a nickel-based cellular material, which has the strength of titanium and the density of water. The material’s strength arises from size-dependent strengthening of load-bearing nickel struts whose diameter is as small as 17 nm and whose 8 GPa yield strength exceeds that of bulk nickel by up to 4X. The mechanical properties of this material can be controlled by varying the nanometer-scale geometry, with strength varying over the range 90–880 MPa, modulus varying over the range 14–116 GPa, and density varying over the range 880–14500 kg/m^3^. We refer to this material as a “metallic wood,” because it has the high mechanical strength and chemical stability of metal, as well as a density close to that of natural materials such as wood.

## Introduction

Cellular materials with spatially organized and repeating pore geometries can have dramatic strength improvements as structural elements shrink to the nanometer scale^1–4^. This paper describes a nanostructured cellular material based on electroplated nickel (8,900 kg/m^3^ bulk density), which has the strength of titanium and the density of water (1,000 kg/m^3^). The high strength arises from size-dependent strengthening of load-bearing nickel struts whose diameter is as small as 17 nm and whose strength is as high as 8 GPa. We refer to this material as a “metallic wood,” because it has the high mechanical strength and chemical stability of metal, as well as a density close to that of natural materials such as wood.

Cellular materials with nanometer-scale structural elements exhibit remarkable material properties^5^, for example high strength^3,6–10^, high energy absorption^4^, ultra-low density^1,11–14^, and high specific strength^1,15,16^. To understand the origin of these enhanced properties, we can look at the performance of single nanopillars which approximate a single strut in a nanostructured cellular material. Compression tests on nanopillars made from single crystal metals showed that reducing pillar diameter increased pillar strength by up to an order of magnitude more than the bulk material’s strength^17–19^. This size-dependent strength enhancement has been widely studied in single crystals^17,18,20–22^. In nanopillars made from nanocrystalline metals, however, both an increase in strength (for Ni and Au)^23,24^ and decrease in strength (for Ni and Pt)^25,26^ have been observed as geometric dimensions were reduced to the size of a single grain diameter. When nanocrystalline gold was used in the struts of a nanostructured cellular material, the cellular solid showed dramatic strength enhancement as the strut diameter decreased^3,6^, but the conflicting results in nanopillars make it difficult to predict if nanostructured cellular materials made from nanocrystalline metals will exhibit a strength enhancement or reduction.

In addition to nanoscale enhancement, the macroscopic properties of nanostructured cellular materials are important when considering them for engineering applications. Most articles on nanostructured cellular materials report specific strength below 100 MPa/(Mg m^3^), which is comparable to polymers and common metals^1,3,4,6–16,27–29^. Materials with specific strength above 100 MPa/(Mg/m^3^) are attractive because their strength approaches that of engineering metals and ceramics^1,15,16^. However, the density of these materials can be low, in the range of 6–410 kg/m^3^, which prohibits their use in some applications. Consider, for example, a metal panel modeled as a simply supported beam with length 1 m, cross-sectional thickness 1 mm, specific strength 100 MPa/(Mg/m^3^), and density 100 kg/m^3^. For an application in which the panel supports a 100 N load at the center, the sheet would require a minimum width of 15 m. In contrast, a panel with specific strength 100 MPa/(Mg/m^3^) and density 1,000 kg/m^3^ would require a minimum width of 1.5 m, which is in general a more reasonable solution. Many structural materials have density near 1,000 kg/m^3^, including engineering polymers and naturally occurring materials such as wood. Only a few articles report nanostructured cellular materials with density near 1,000 kg/m^3^, and these articles focus on ceramics and carbon based materials rather than metals^4,16,29,30^. In general, there is a lack of published research on strong nanostructured cellular materials that have enough mass to support loads found in industrial applications.

Here we report a cellular material based on nanostructured nickel inverse opal materials. The mechanical properties of this material are governed by the size-dependent strengthening of nanometer-scale structural elements, allowing large specific strengths up to 230 MPa/(Mg/m^3^) in porous nickel. This specific strength is larger than most high strength metals including high strength stainless steel and Ti-6Al-4V^31,32^. The mechanical properties of this material can be varied between natural materials and high strength metal alloys by controlling the geometric parameters within the cellular architecture; we demonstrate materials with yield strengths over the range 90–880 MPa, specific moduli 7–25 GPa/(Mg/m^3^), and densities 880–14500 kg/m^3^. Using finite element simulations and well established micropillar compression and nanoindentation testing, we find that the material strength increases as the nanometer-scale strut diameter decreases. The strut yield strengths increase from 3.8 to 8.1 GPa as the strut diameter decreases from 115 to 17 nm, which is a 4X increase over the 2 GPa bulk deposited nickel yield strength.

## Results

**Figure 1:**
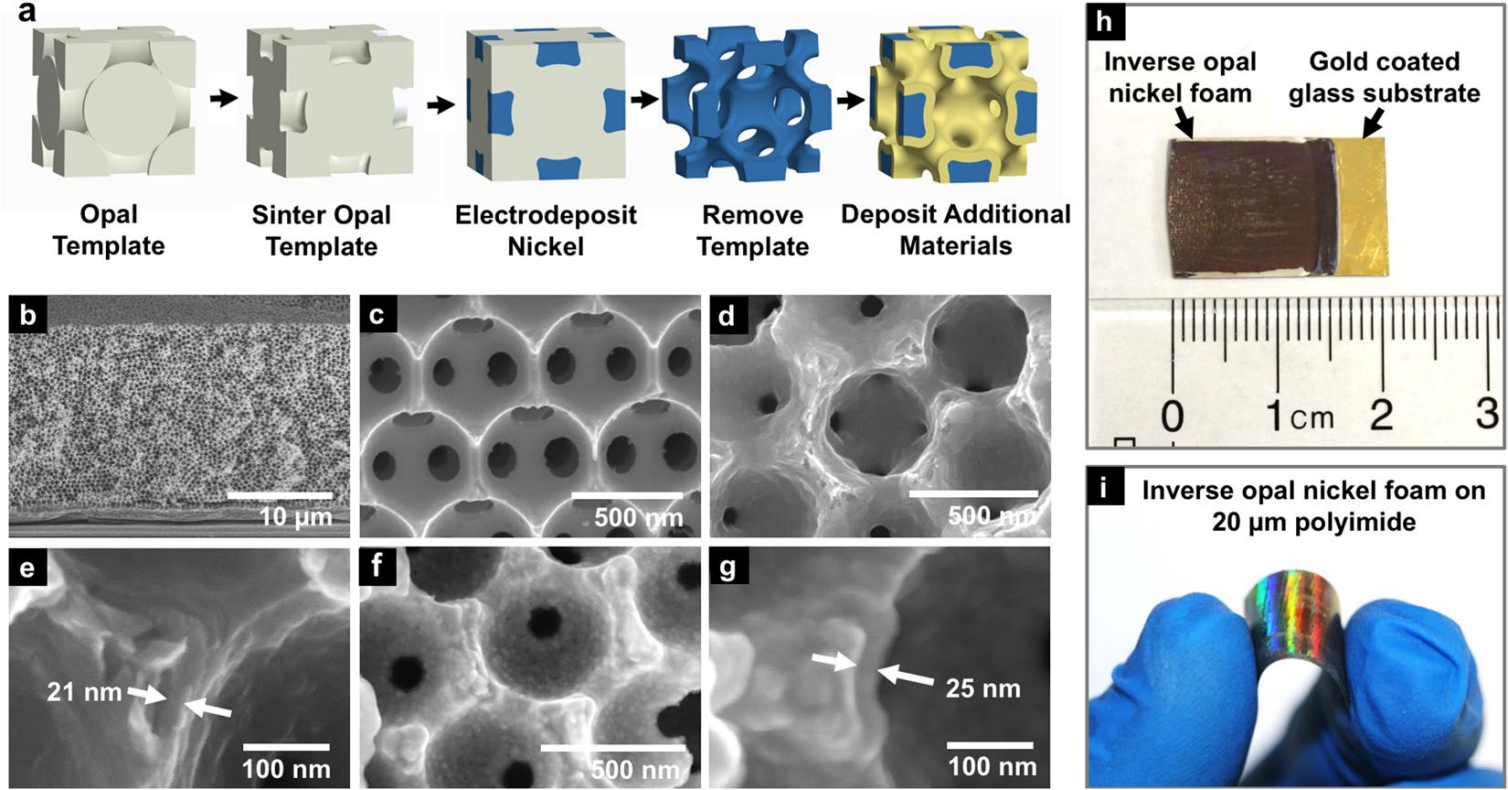
(**a**) The fabrication process for a unit cell of the nickel inverse opal material. (**b**–**g**) Cross section SEM images of nickel inverse opal material. (**b**,**c**) A nickel inverse opal with no coating. (**d**,**e**) A nickel inverse opal material with a 21 nm coating of additional electrodeposited nickel. (**f**) A nickel inverse opal material with a 25 nm coating of additional electrodeposited rhenium-nickel. (**g**) A closer image of one of the struts in (**f**). (**h**) A 2 cm^2^ nickel inverse opal material with 500 nm pores and 15 µm thickness grown on a gold/chromium coated glass slide. (**i**) A nickel inverse opal material with 300 nm pores grown on gold/chromium coated 20 µm thick polyimide.

Figure 1a shows the fabrication of the inverse opal material. First, monodisperse polystyrene (PS) particles of diameter 260–930 nm were self-assembled onto a gold/chromium coated substrate in a face centered cubic (FCC) orientation. The PS was sintered at 96 °C to improve stability and increase the interconnect diameter between PS spheres. Nickel, 99.9%, was electrodeposited into the voids of the PS structure, followed by PS etching in tetrahydrofuran. The result was an open cell inverse opal material having interconnected spherical pores in a face-centered cubic orientation. For some samples, conformal electrodeposition of either additional nickel or rhenium-nickel alloy (80 wt% rhenium) increased the solids volume fraction, strut diameter, and mass. The additional deposited nickel had the same composition as the nickel used to fabricate the inverse opal structure. Mechanically tested samples with rhenium are labeled Re. The average grain sizes of the nickel film and nickel inverse opals were 12.4 nm and 15.1 nm, calculated from the XRD data shown in the supplementary information using the Scherrer equation. The grain size was similar for coated and un-coated nickel samples. A 15 nm grain size in pure nickel corresponded to a ≈6.4 GPa hardness^33,34^, near the peak hardness values of pure nickel, which agreed well with our electrodeposited nickel thin film nanoindentation hardness measurements of 5.8 GPa. The self-assembly based fabrication method can produce material samples larger than 100 mm^2^, while allowing ~10 nm control of structure and chemistry by altering the polystyrene diameter, processing temperature, and electroplating parameters. In comparison, most methods for fabricating nanostructured cellular solids with controlled and repeated unit cell morphologies are limited to sample sizes with areas smaller than about 5 mm^2^ ^1,11,12,15,27,28,35^. Figure 1b–g show SEM images of the material with 500 nm pores fractured on the (111) plane. Figure 1b,c show the inverse opal material with no coating and 0.16 solids volume fraction. Figure 1d,e show the material uniformly coated with 21 nm of nickel, which increased the solids volume fraction from 0.16 to 0.35. Figure 1f,g show the nickel inverse opal material uniformly coated with 25 nm of rhenium-nickel alloy, which increased the solids volume fraction to 0.46. Figure 1h shows a material with 500 nm pores, 2 cm^2^ area, and 15 µm thickness fabricated on a gold/chromium coated glass slide. Figure 1i shows a material with 300 nm pores and 20 µm thickness fabricated on gold/chromium-coated polyimide. The material is flexible and when prepared on a polyimide sheet could be bent past a 0.5 cm radius (Fig. 1i). Table 1 shows the fabricated materials and their geometric attributes such as coating thickness and volume fraction.

**Table 1:** Mechanical and physical measurements of inverse opal cellular materials.

Material	Pore size [nm]	Coating thickness [nm]	Smallest effective diameter [nm]	Total solids volume fraction	Density [g cm^-3^]	Hardness [GPa]	Modulus [GPa]	Yield strength* [GPa]	Strut yield strength [GPa]	Specific strength* [MPa/(Mg/m^3^)]	Specific modulus [GPa/(Mg/m^3^)]
Ni Bulk				1.00	8.9	5.806	171	1.98		222	19.2
Ni IO**	260		16	0.10	0.9	0.213	17				18.7
Ni IO	260		17	0.10	0.9	0.218	17	0.19	8.07	220	19.3
Ni IO	500		46	0.19	1.7	0.321	34				20.0
Ni IO	470		41	0.15	1.3	0.255	25				19.1
Ni IO	520		51	0.16	1.4	0.315	29	0.27	5.61	190	20.1
Ni IO	500		48	0.23	2.1	0.359	30				14.7
Ni IO	500		45	0.28	2.5	0.316	27				10.8
Ni IO	470		45	0.21	1.9	0.374	27				14.9
Ni IO	490		48	0.18	1.6	0.259	21				13.3
Ni IO	930		59	0.10	0.9	0.082	14	0.09	3.82	102	15.9
Re on Ni IO	520	5		0.21	2.3	0.508	51				22.0
Re on Ni IO	500	8		0.34	3.9	0.499	44				11.3
Re on Ni IO	490	15		0.32	4.1	1.024	71				17.3
Re on Ni IO	500	18		0.36	4.8	0.977	48				10.0
Re on Ni IO	490	19		0.34	4.4	0.953	51				11.6
Re on Ni IO	500	25		0.46	6.6	1.236	61	0.69	2.88	168	9.2
Re on Ni IO	500	41		0.57	9.2	1.386	70				7.6
Re on Ni IO	470	59		0.57	10	2.870	100				10.0
Re on Ni IO	470	87		0.72	15	3.950	116				7.7
Ni on Ni IO	500	5	57	0.27	2.4	0.413	42				17.4
Ni on Ni IO	500	13	74	0.34	3.0	0.706	59				19.7
Ni on Ni IO	470	19	83	0.35	3.1	1.118	70	0.61	3.86	197	22.5
Ni on Ni IO	470	25	93	0.39	3.5	1.193	70				20.0
Ni on Ni IO	495	28	120	0.39	3.5	1.672	85				24.8
Ni on Ni IO	495	33	115	0.43	3.8	2.206	95	0.88	4.07	229	24.8
Ni on Ni IO	520	38		0.48	4.3	2.379	74				17.3

*Data from micropillar compression tests.

**IO: inverse opal.

The high specific strength of the inverse opal material results from two mechanical processes: porosity-based weakening, which is independent of size, and strengthening of the pore struts, which is size-dependent at the nanometer-scale. Porosity-based weakening is the reduction in load that the material can support due to reduced volume fraction of solid material, and mediated by bending dominated deformation of the material struts^36^. Size-dependent strengthening increases the local strength of the constituent material because of dislocation starvation in the struts, for strut widths smaller than about 1 µm^2,9,19,37^. The inverse opal material yield strength, σ^*^, is related to the solids volume fraction, (ρ^*^/*ρ*_*s*_), and strut yield stress, *σ*_*y*_, by

1 $${\sigma }^{\ast }={C}_{1}\,{\sigma }_{y}(d){(\frac{{\rho }^{\ast }}{{\rho }_{s}})}^{{C}_{2}},$$

where *σ*_*y*_ depends on the effective strut diameter, *d*, and can be greater than the bulk material strength due to size-dependent strengthening, ρ^*^ is the density of the porous inverse opal, and *ρ*_*s*_ is the density of the bulk solid^8,36^. The geometric coefficients, *C*_1_ and *C*_2_, are unique to the inverse opal geometry and independent of both pore size and solid chemistry. Once *C*_1_ and *C*_2_ are known for a given lattice geometry, the size-dependent strengthening effect *σ*_*y*_(*d*) can be determined by measuring σ^*^, *d*, and ρ^*^/*ρ*_*s*_^3,8,9^.

**Figure 2:**
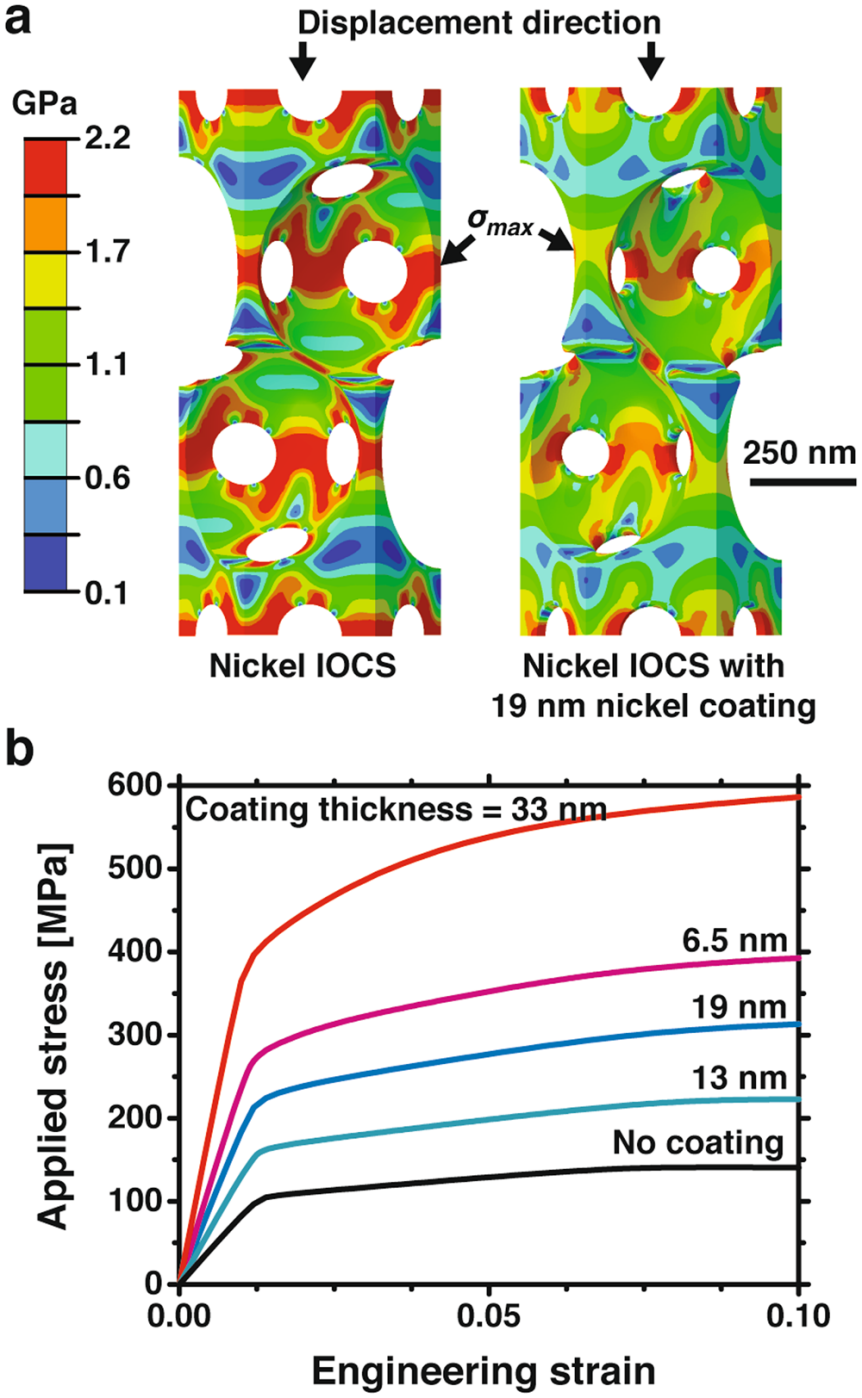
(**a**) Unit cells of the nickel inverse opal material used to determine the yield strength and elastic modulus through finite element simulations. The unit cell on the right has an additional 19 nm nickel coating. The colors correspond to Von Mises stress at a 15 nm displacement, near the yield point. (**b**) Calculated stress-strain curves of nickel inverse opal material with 0–33 nm thick nickel coatings.

We used finite element simulations of an inverse opal unit cell to understand how the geometry relates to the material modulus and yield strength and to solve for *C*_1_ and *C*_2_ (Fig. 2). Finite element simulations improved upon previous work, which used idealized relationships to calculate the size-dependent strengthening in nanoporous gold^3,8,9^, by more accurately relating the applied load to the deformation and stress distribution in the inverse opal geometry. The simulations considered an inverse opal unit cell with isotropic elasticity and J2 plasticity loaded in the [111] direction. In order to mimic the experiments and control the solids volume fraction, the simulations included 0–33 nm nickel coatings, where the coating has the same material properties as the base nickel. Figure 2a shows repeat unit cells used in the finite element simulations; the colors correspond to Von Mises stresses at 1.2% engineering strain. The maximum stress occurs in the narrow region of the struts parallel to the displacement direction. Figure 2b shows corresponding stress-strain curves for nickel inverse opals with various coating thicknesses. The yield strengths, σ^*^, were calculated at a 0.2% strain offset and compared to the solids volume fraction to solve for the coefficients in Eq. 1 ^38^. The cellular solid yield strength is

2 $${\sigma }^{\ast }=0.78\,{\sigma }_{y}{(\frac{{\rho }^{\ast }}{{\rho }_{s}})}^{1.52}.$$

For each material, the elastic modulus, E^*^, was calculated from the initial slope of the linear stress-strain region in Fig. 2b. The elastic modulus is related to the solids volume fraction and bulk nickel elastic modulus, *E* = 171 GPa, by

3 $${E}^{\ast }=1.0\,E{(\frac{{\rho }^{\ast }}{{\rho }_{s}})}^{1.73}.$$

The polynomial relationship between the mechanical properties and solids volume fraction indicates that the cellular solid deforms primarily through bending^39^. This bending-dominated deformation is expected, since the inverse opal cell, with 32 struts and 21 joints, does not satisfy the Maxwell criterion^40^. The stress and modulus relations compare closely with measurements described below, and are valid for coated and un-coated inverse opals made from any one material.

**Figure 3:**
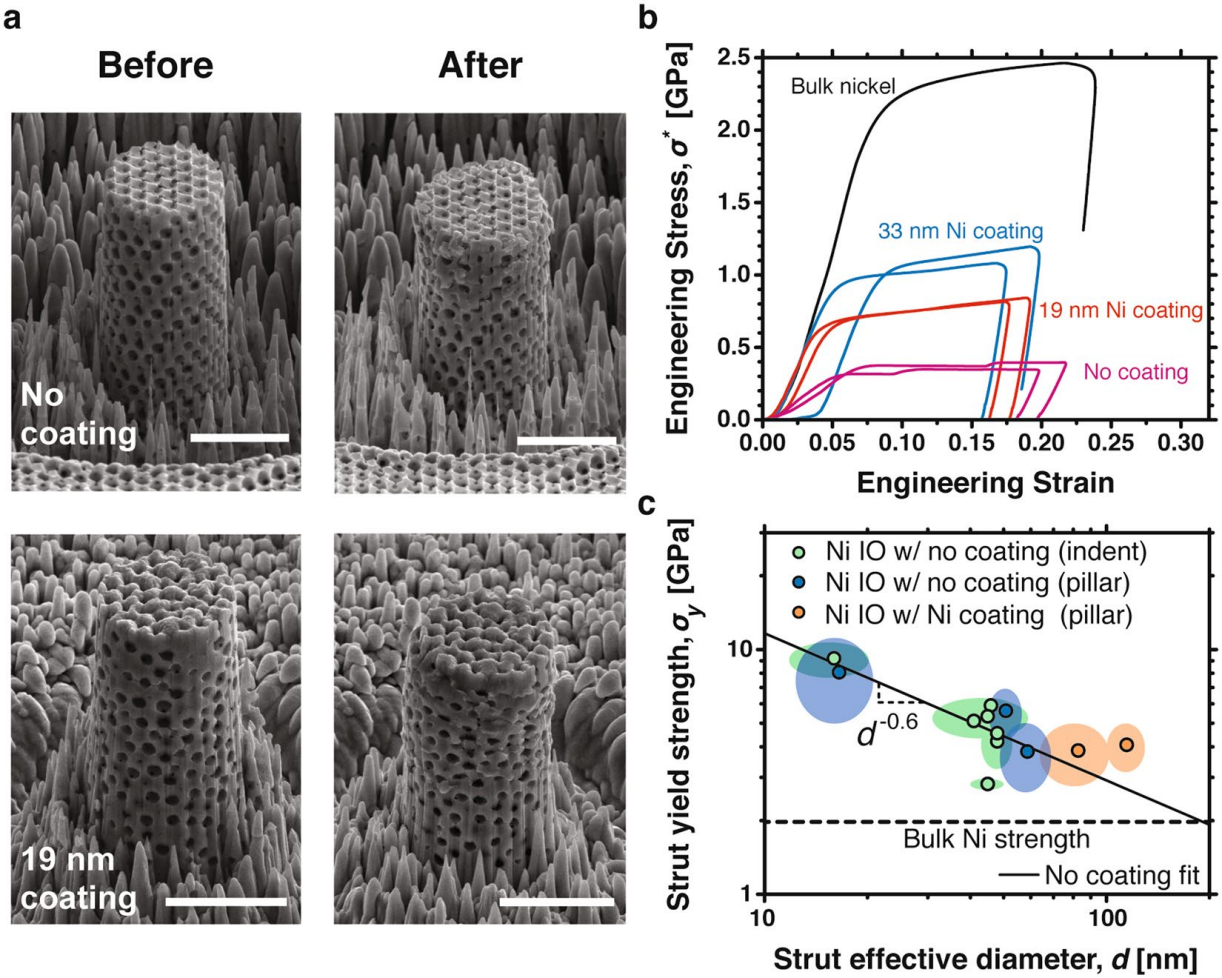
(**a**) SEM images of inverse opal micropillars with 500 nm pores, before and after compression testing. The uncoated sample is 84% porous and the 19 nm nickel coated sample is 58% porous. Failure occurs in the [111] direction, parallel to the compression axis. Scale bars are 3 µm. (**b**) Engineering stress, σ^*^, versus engineering strain for micropillars under compression. The nickel sample is bulk electroplated nickel. (**c**) Strut yield strength, *σ*_y_, as a function of strut diameter, *d*, for nickel inverse opal materials with 260, 500, and 930 nm pores. Blue data are nickel inverse opals measured with micropillar compression tests. Green data are nickel inverse opals measured with nanoindentation. Orange data are 500 nm pore nickel inverse opals coated with 19 and 33 nm of additional nickel. Shaded regions represent standard deviations. The fit line was only applied to nickel samples with no coatings.

We measured mechanical properties of the fabricated materials using nanoindentation and micropillar compression measurements, which are well established techniques for measuring nanostructured cellular material compressive strengths and moduli^1,3,4,6–19^. The micropillar compression tests measured the yield strength, σ^*^^17^, while nanoindentation measurements allowed us to determine elastic modulus, E^*^, and hardness for all materials. Figure 3 shows micropillar compression test results for materials made from 500 nm diameter PS particles and 0, 19, and 33 nm nickel coatings. Figure 3a shows SEM images of 4 µm diameter micropillars before and after compression tests. Failure predominately occurred at the narrowest region of the struts and in the [111] direction parallel to the micropillar axis, consistent with the finite element simulations, which are orientated in the same geometry. Figure 3b shows the measured engineering stress-strain data for several inverse opal and solid nickel micropillars loaded to an engineering strain of about 20%. Table 1 shows the measured yield strength, which was measured using the 0.2% offset method.

The measured micropillar yield strength, σ^*^, provides insight into the size-dependent strength of the inverse opal struts, *σ*_y_(*d*). The supplementary methods section describes measurements of effective strut diameter, *d*, and solids volume fraction, (ρ^*^/*ρ*_*s*_), which are combined with Eqn. 2 for this analysis. Figure 3c shows strut yield strength, *σ*_y_, as a function of effective strut diameter at the narrowest region of the strut, approximately where the failure occurred, for nickel inverse opals with 260, 500, and 930 nm pores. Blue data points show the strength of nickel inverse opals measured with micropillar compression. Green data points are nickel inverse opals measured with nanoindentation, where the yield strength was approximated by the hardness. Orange data points are 500 nm pore nickel inverse opals coated with 19 and 33 nm of additional nickel and measured with micropillar compression. In general, the strut strength increased as the effective strut diameter decreased. The largest value of strut yield strength measured by micropillar compression was 8.07 GPa for the nickel inverse opal material with 260 nm pore size, 0.10 solids volume fraction, and 17 nm effective strut diameter. This strength was a 4X increase over the yield strength of bulk electrodeposited nickel, which is 1.98 GPa^33^. Importantly, the 1.98 GPa yield strength of the bulk nanocrystalline nickel was near the maximum reported for pure nickel^33,34^, so the large strength enhancement must be due to the pore structure introduced by the inverse opal geometry. Similar strengthening has previously been reported in solid single crystal micro and nanopillar compression experiments, where the strength of sub-μm diameter pillars approached the theoretical strength of the material^2,9,19,20,23,37,41,42^, as well as in nanoporous gold and mesoporous copper lattices with nanocrystalline and polycrystalline microstructure^3,8,28^. The −0.6 slope of the best fit line for the uncoated nickel samples agreed remarkably well with the approximately −0.6 slope experimentally measured in multiple single crystal FCC metals including nickel, aluminum, gold, and copper^17^, as well as with the −0.66 slope measured in nanocrystalline nickel nanopillars^24^. This good agreement indicates that the strengthening mechanism and initial dislocation density in the nickel inverse opal samples were similar to previous single crystal and nanocrystalline nanopillar samples. The strengthening is derived from the small grain or feature size, which leaves no mechanism for dislocation build up and the dominant form of dislocation nucleation becomes surface nucleation^24^. Similar to single crystal materials, the smaller strut diameters are stronger because the decreased probability of a critical dislocation compared to larger struts and bulk materials^24^. The results in Fig. 3c show a clear size-dependent strength enhancement in inverse opal materials.

Figure 3b and Table 1 show that the inverse opal strength increased in nickel inverse opals coated with additional nickel. The inverse opal strength increase is due to the strong dependence of the material strength on relative density (Eqn. 2), despite the strut yield strength decreasing as the strut diameter increased (Fig. 3c). Interestingly, the strut yield strength exceeds the strength predicted by the fit line in Fig. 3c, which indicates that coatings could provide a method for enhancing the total strength of inverse opal composites. Currently, the exact mechanism for the strength enhancement and the interaction between coated layers is not clear.

**Figure 4:**
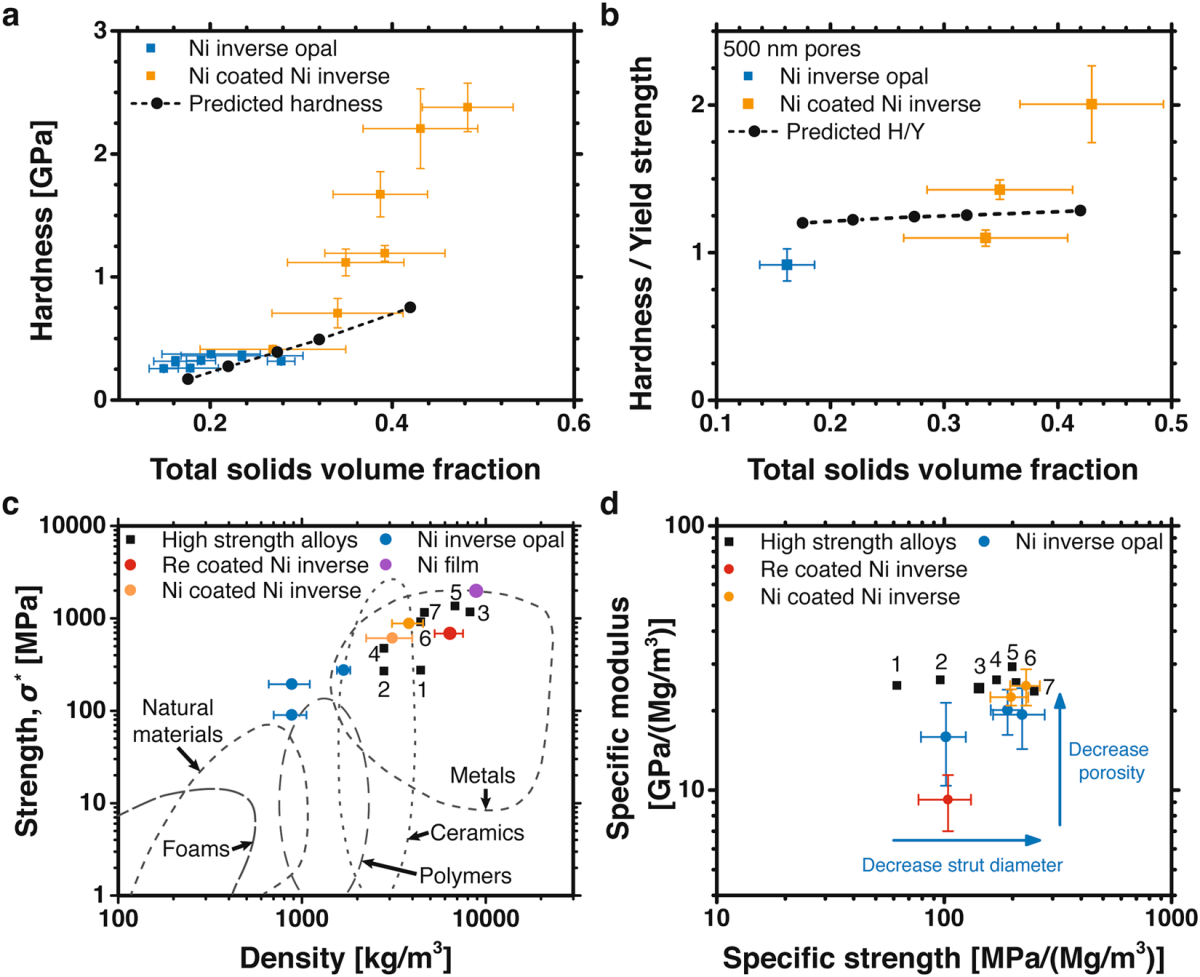
(**a**) Nanoindentation hardness measurements versus total solids volume fraction of the inverse opal materials. The black dots are numerical predictions of the hardness measurements from finite element simulations. (**b**) Nominal hardness (hardness normalized by the yield strength) versus total solids volume fraction of several uncoated and nickel-coated nickel inverse opal materials. The flow stress at 10% strain is used as the yield strength for the nominal hardness measurements. The black dots are numerical predictions of the nominal hardness. (**c**) Ashby plot of material strength, calculated at a 0.2% strain offset, versus density for the fabricated materials and several reference materials. (**d**) Ashby plot of specific modulus and specific strength of the fabricated materials and several reference materials. For comparison in (**c**) and (**d**), common Ti, Al, Ni, and Fe high strength alloys are labeled as follows 1 - CP Ti, 2 - 2024-T4, 3 - Inconel 718, 4 - 7075-T6, 5 - HSSS steel, 6 - Ti-6Al-4V, 7 - Ti-10V-2Fe-3Al^31,32,67,68^. Error bars show standard deviations.

The high strength of the inverse opal struts combined with the regular porous architecture imparts unique macroscopic material properties including high hardness, high strength at a given weight, and a controllable modulus while maintaining high specific strength. Figure 4 shows inverse opal material properties and compares them to other high strength engineering materials. Figure 4a shows hardness as a function of solids volume fraction for nickel and nickel-coated nickel inverse opal materials. Hardness measures the resistance to local plastic deformation and is important for several applications including tribology. The hardness was measured with nanoindentation and increased from 0.082 to 2.4 GPa as the solids volume fraction increased from 0.1 to 0.48. The most dramatic hardness increase, from 0.3 to 2.4 GPa, occurred between 0.28 and 0.48 solids volume fractions, where the inverse opal hardness approached the linear scaling of the bulk hardness and solids volume fraction. The hardness predicted from finite element simulations of the nanoindentation tests are shown in black and have good agreement with the measured hardness at 0.2 to 0.3 solids volume fractions^41,43^; however, the simulations under predict the hardness at low (less than 0.2) and high (greater than 0.3) solids volume fractions. Nanoindentation simulation details can be found in the supplementary methods section. The larger than predicted material hardness at high solids volume fractions results from a larger than predicted plastic Poisson’s ratio. This can be seen by comparing the measured and calculated nominal hardness, hardness normalized by yield strength, which depends strongly on the plastic Poisson’s ratio (Fig. S2d). Figure 4b shows that the measured and predicted nominal hardness both increase with solids volume fraction, but the measured nominal hardness increases dramatically at greater than 0.35 solids volume fractions while the predicted nominal hardness remained ~1.3. This jump in the measured nominal hardness is likely due to rapid densification of the inverse opal geometry during large plastic strains, which would increase the plastic Poisson’s ratio and increase the nominal hardness. Interestingly, the dramatic hardness increase in inverse opal materials with greater than 0.3 solids volume fractions corresponds to the same volume fractions where previous simulations of inverse opal materials predicted a rapid increase of modulus towards the Hashin and Shtrikman upper bound for isotropic materials^29,44,45^. At greater than 0.3 solids volume fraction, simulations show that the inverse opal structure can outperform the state-of-the-art octet- and isotropic-truss geometries in isotropic Young’s modulus, shear and bulk modulus, as well as in structural efficiency (total stiffness)^16,29^. At smaller than 0.3 solids volume fraction, the inverse opal hardness is a good predictor of the yield strength.

Figure 4c shows the strength versus density of our materials along with data from other materials for comparison purposes, including high strength Ti, Ni, Al, and steel alloys. The nickel inverse opal materials with no coatings had the strength of aluminum alloys and titanium (σ^*^ = 90–240 MPa), and density close to that of water (ρ^*^ = 880 to 1400 kg m^−3^), filling a new material space in the strength-density relationship. The two lightest inverse opal materials had the same average solids volume fractions ((ρ^*^/*ρ*_*s*_) = 0.1); however, the sample with smaller effective strut diameters (*d* = 17 vs. 59 nm) had an increased strut yield strength (*σ*_*y*_(*d*) = 10 vs. 4.6 GPa) that more than doubled the material yield strength (σ^*^ = 194 vs. 90 MPa). The presence of a coating further increased the material strength. The strength and density of the coated inverse opal materials was comparable to high-strength, lightweight alloys such as 7075-T6 aluminum, Ti-6Al-4V titanium, and Ti-10V-2Fe-3Al titanium. These material properties were achieved despite the inverse opal material being fabricated from dense engineering materials (Ni = 8,900 kg m^−3^ and Re = 21,020 kg m^−3^).

The unique relationship between the strut strength and inverse opal geometry enables independent control of the specific modulus and specific strength. Figure 4d shows the specific strengths versus specific moduli of the nickel inverse opal materials. The average specific strengths varied between 102–230 MPa/(Mg/m^3^) and the average specific elastic moduli varied between 9–25 GPa/(Mg/m^3^). Uncoated nickel inverse opal materials had solid volume fractions between 0.10 and 0.28 and average specific moduli between 15 and 20 GPa/(Mg/m^3^). Coating additional layers of nickel onto the inverse opal materials increased the solids volume fractions up to 0.48 and increased the average specific moduli to 25 GPa/(Mg/m^3^). In some cases, the specific moduli exceeded the bulk nickel specific modulus (19.2 GPa/(Mg/m^3^)), likely due to the non-negligible contribution of the stiff native oxide. As additional layers of nickel were coated, the specific strengths remained near 200 MPa/(Mg/m^3^) because an increased solids volume fraction increased the geometric strength but also increased the strut diameter, which reduced the benefit of size-based strengthening. As described by the arrows in Fig. 4d, the specific modulus can be controlled independent of the specific strength by increasing the total solids volume fraction; whereas, the specific strength can be independently controlled by changing the strut effective diameter while maintaining a constant solids volume fraction.

## Discussion

The inverse opal geometry enables unique mechanical properties. The specific strengths reported here and by Do Rosario *et al*.^16,29^ are greater than the specific strengths reported for nanoscale octet-truss architectures at similar volume fractions (10–150 MPa/(Mg/m^3^)), which ideally deform with no bending^1,12,27^. We hypothesize that the high specific strength of inverse opal materials arises from the large concentration of ultra-high strength struts that bear the majority of the stress concentrations, and the smooth transitions between struts that constrain bending modes and reduce stress concentrations where the struts connect. For example, and inverse opal material with 500 nm pores has 32 struts in every 0.35 µm^3^. The high strength narrow regions in the struts support a majority of the stress concentrations and are smoothly connected to each other at ~100 nm thick nodes that prevent twisting and stress concentrations where the struts connect (Fig. S1). This pore geometry resembles closed-cell foams with small interconnected openings between pores; closed-cell foams outperform open-cell materials because the cell faces constrain the bending modes of cell edges^29,44^. Defects or non-closed-packing of particles in the self-assembly process caused disorder and increased the strut diameters and solids volume fraction, which decreased specific strength. Well-ordered inverse opal materials have a unique geometry that enables high specific strengths.

The specific strength of these materials could be further improved by fabricating smaller struts at increased volume fractions, and also with fewer defects in the crystal structure. For example, a perfectly ordered nickel inverse opal with 0.3 solids volume fraction and <10 nm effective strut diameters could achieve a specific strength of 520 MPa/(Mg/m^3^) because the strut strengths would be close to the ~10.5 GPa theoretical yield strength of nickel^46^. By using lightweight metal inverse opals such as titanium and aluminum, it might be possible to achieve 1,463 and 770 MPa/(Mg/m^3^) specific strengths at 0.3 solids volume fractions as their theoretical yield strengths are ~15 and ~4.7 GPa^47,48^.

## Conclusion

In conclusion, we present metallic wood fabricated from nickel inverse opals, which has the strength of titanium and the chemical properties of a metal, while having the density of water and the cellular nature of natural materials like wood. The high strength of the metallic wood results from the size-dependent strengthening of the inverse opal struts, which have up to 4X the yield strength of bulk electrodeposited nickel and enable high specific strengths of 230 MPa/(Mg/m^3^). The cellular structure can be controlled to tune the modulus and strength each by a factor of 10X.

The metallic wood can be easily fabricated over 100 mm^2^ areas, can be processed at room temperature, and can be combined with additional functional materials, as demonstrated with the rhenium coatings^49^. The high strength continuous metallic architecture with isotropic elasticity, high hardness, and high strain energy storage could be important for a variety of applications such as energy storage^50–52^, heat transport^53^, and sensors^54,55^. Future work could explore improvements in specific strength above 230 MPa/(Mg/m^3^) by incorporating lightweight metals such as titanium or aluminum and developing roll-to-toll processing of high strength porous metals from self-assembly^56–58^.

## Methods

### Fabrication

The inverse opal cellular solids were fabricated by self-assembling polystyrene (PS) opals onto a gold-coated glass slide, electrodepositing nickel, and etching the remaining PS. The gold coated glass slide was fabricated by sputtering 8 nm of chromium followed by 50 nm of gold on a 1 mm-thick soda lime glass slide. The glass slide with a gold film was cut into smaller samples, piranha cleaned for ten minutes, immersed in Millipore water with 3-mercapto-1-propanesulfonic acid and sodium salt (2.2% by weight) for 4 hours and rinsed. Polystyrene (PS) opals were self-assembled onto the gold coated substrates by placing the substrates vertically in a 1 inch diameter plastic container filled with a colloidal solution of PS spheres. The plastic container was set on a hot plate at 55 °C, covered, and left for 24 to 30 hours until the solution was dry. The PS diameters were varied between 200–2,000 nm to change the cellular solid strut size. The substrates were then sintered at 96 °C for 30 minutes to 6 hours depending on the PS diameter. Longer sinter times increased the interconnect diameter between spheres and reduced the nickel volume fraction. The PS colloidal solution was made by combining 8 wt% PS sphere solution (1.2 grams), purchased from Invitrogen, with ultrapure water (40 grams). Nickel was then electrodeposited through the PS opal at a constant −1.8 volts versus a nickel reference electrode for 32 minutes in commercial plating solution, Technic RTU Mechanical Agitation. PS was removed by immersing the substrates in a tetrahydrofuran bath for 24 hours followed by a tetrahydrofuran and toluene rinse. The resulting cellular solids were about 15–20 µm thick. Additional nickel was coated on the inverse opal cellular solids using the same electroplating solution, but pulsing −1.7 volts for 30 seconds in between 20 second intervals of 0 amperage current for 15–90 cycles. Pulsed plating allowed for uniform nickel coating throughout the inverse opal depth^52^. The nickel coating had a standard deviation between 10 and 30 percent of the average coating thickness for all samples. This variation was measured and accounted for in all calculations.

Rhenium-nickel alloy was used as a coating because of its high ductility, strength, and creep resistance at high temperatures^59^, which has made it an interesting material for thermophotovoltaics^49^. The rhenium-nickel alloy coating was deposited galvanostatically with a 5 mA cm^−2^ current density in a pH 5 electroplating bath with 34 mM NH_4_ReO_4_, 93 mM Ni(NH_2_SO_3_)_2_•4H_2_O, and 300 mM C_6_H_8_O_7_ modified from ref.^60^. NaOH was used to adjusted the pH. ReO_4_^−^ is the most stable form of rhenium ion in solution. Citric acid, a tri-basic acid, deprotonates gradually as the pH is increased and is a complexing agent. The plating bath was immersed in silicone oil at 75 °C. A platinum reference and counter electrode was used. Varying the precursor concentration changed the alloy composition. The plated alloy was about 80 weight percent rhenium measured with energy-dispersive X-ray spectroscopy.

### Material characterization

The mechanical properties of the bulk materials depend on their crystal structure. Figure S1f shows XRD data for a solid nickel film, a nickel inverse opal, and a solid rhenium film deposited on tungsten. The average grain sizes of the nickel film and nickel inverse opals were 12.4 nm and 15.1 nm, calculated from the XRD data using the Scherrer equation. A 15 nm grain size in pure nickel corresponded to a ≈6.4 GPa hardness^33,34^, near the peak hardness values of pure nickel, which agreed well with our electrodeposited nickel thin film nanoindentation hardness measurements of 5.8 GPa. The deposited rhenium-nickel alloy was amorphous with some polycrystallinity detected in transmission electron microscopy diffraction patterns.

The nickel inverse opal solids volume fraction, (ρ^*^/*ρ*_*s*_)_*Ni*_, and coating volume fraction, (ρ^*^/*ρ*_*s*_)_*coat*_, are the independent variables for calculating the effective mechanical properties of cellular solids. (ρ^*^/*ρ*_*s*_)_*Ni*_ and (ρ^*^/*ρ*_*s*_)_*coat*_ are calculated from SEM measurements of PS radius, *R*, interconnect diameter, *b*, and coating thickness, *t*, combined with a geometric model of self-assembled PS spheres organized in a FCC unit cell, details of which are in the supplementary methods.

### Mechanical simulations

Uniaxial compression tests were simulated using the implicit version of the commercial finite element package ABAQUS by modeling a three-dimensional unit cell of the inverse opal material with ten-node nonlinear tetrahedron elements. Since the inverse opal material was subjected to a macroscopically homogeneous stress state during the uniaxial compression test, it was sufficient to consider a unit cell to evaluate the effective mechanical properties. Figure S2a shows the hexagonal unit cell orientation, chosen to best represent the inverse opal geometry in the nanoindentation and micropillar compression tests. The unit cell was modeled with isotropic elasticity and J2 plasticity using the measured properties of the electrodeposited nickel. A displacement boundary condition was applied normal to the top surface and the average reaction force at the displaced boundary was recorded at each displacement increment^61,62^. The elastic modulus of cellular structures was calculated at the first increment of simulations. The flow stress at 0.2% offset plastic deformation was chosen as the yield strength. Additional details on the simulation setup and boundary conditions are provided in the supplementary methods.

We used a numerical method for simulating the Berkovich nanoindentation test. The main aim of the nanoindentation study was to determine the nominal hardness, $\frac{H}{Y}$, which is the ratio of the hardness, *H*, to the flow strength, *Y*. Figure S2b shows the simplified indentation problem. We represented the three-dimensional Berkovich tip as a conical indenter with half cone angle *θ* = 70.3°^63^, so that the nominal contact area *A*_*n*_ was the same as the Berkovich tip. The conical indenter was forced into the center of a cellular solid material block of size *L* at the center (Fig. S2b). As a result, only the axisymmetric mode of deformation was considered, and the corresponding boundary value problem was solved in cylindrical coordinates (*r*, *z*).

The cellular solid block bottom was fixed in the *z* direction, *u*_*z*_|_*z* = 0_ = 0, while a symmetric boundary condition was imposed on the center line, *u*_*r*_|_*r* = 0_ = 0. The indenter was assumed to be rigid and frictionless, and was statically driven into the block with displacement *h*. We evaluated the reaction force, *F*(*h*), and the nominal contact area, *A*_*n*_(*h*) = *πa*(*h*)^2^. The hardness, *H*, at indentation depth *h* was calculated as

4 $$H(h)=\frac{F(h)}{\pi a{(h)}^{2}}.$$

The boundary value problem was solved using the implicit version of the commercial Finite Element Package ABAQUS, with 4 nodes axisymmetric elements. To determine the nominal hardness, $\frac{H}{Y}$, the flow strength, *Y*, was chosen as the flow strength at 10% axial strain and kept as a constant. We chose the flow strength at 10% axial strain because, as shown in Fig. 3b, the uniaxial compression response of the cellular solid showed a mild strain hardening behavior after reaching the yield stress, *σ*_*Y*_.

We considered the cellular solid as a homogenized continuum during the indentation process, as the contact radii was large compared to the unit cell size over most of the range of indentation depth *h*. Isotropic elasticity was used to model the elastic behavior of the cellular solid, with Young’s modulus, *E*, and elastic Poisson’s ratio, *υ*^*e*^, calculated from the unit cell compression simulations. Following Khaderi *et al*.^41^, the plastic deformation of the cellular solid was described by the isotropic crushable foam model of Deshpande and Fleck^43^.

The isotropic yield surface of the cellular solid is specified by

5 $$\hat{\sigma }-Y({\hat{\varepsilon }}_{P})=0.$$

The equivalent stress, $\hat{\sigma }$, was composed of the mean stress, *σ*_*m*_, and the Mises effective stress, *σ*_*e*_, by

6 $${\hat{\sigma }}^{2}=\frac{1}{1+{(\frac{\alpha }{3})}^{2}}({\sigma }_{e}^{2}+{\alpha }^{2}{\sigma }_{m}^{2}),$$

where ${\sigma }_{m}=\frac{1}{3}{\sigma }_{kk}$, and ${\sigma }_{e}^{2}=\frac{3}{2}{\sigma ^{\prime} }_{ij}{\sigma ^{\prime} }_{ij}$ with ${\sigma ^{\prime} }_{ij}={\sigma }_{ij}-\frac{1}{3}{\sigma }_{kk}$. The typical compressive response of a cellular solid is characterized by a plateau stress ${\sigma }_{Y}^{pl}$ followed by densification due to the contact between the cell walls at large deformation. Following Khaderi *et al*. we assumed the yield surface $Y({\hat{\varepsilon }}_{P})$ to have the following form^41^

7 $$Y({\hat{\varepsilon }}_{P})=\{\begin{array}{ll}{\sigma }_{Y}^{pl} & {\hat{\varepsilon }}_{P}\le 0.5\\ {\sigma }_{Y}^{pl}+{h}_{d}({\hat{\varepsilon }}_{P}-0.5) & {\rm{otherwise}}\end{array},$$

where the hardening rate *h*_*d*_ after densification was assumed to be equal to the effective Young’s modulus, *E*, of the inverse opal cellular solid. The parameter *α* determined the plastic compressibility by specifying the ratio of deviatoric stress to hydrostatic strength. Assuming the associate flow rule, *α* was determined by evaluating the plastic Poisson’s ratio, *v*_*p*_, through the following relationship,

8 $${v}_{p}=\frac{\frac{1}{2}-{(\frac{3}{\alpha })}^{2}}{1+{(\frac{3}{\alpha })}^{2}}.$$

With *v*_*p*_ = 0.5, plastic incompressibility was maintained, and *v*_*p*_ < 0.5 means the plastic flow was compressible. We thus calculate the plastic Poisson’s ratio from the uniaxial compression simulations with

9 $${v}_{p}=\frac{{\dot{\varepsilon }}_{11}^{p}}{{\dot{\varepsilon }}_{33}^{p}},$$

and hence the corresponding *α* value is obtained. Figure S2c shows the finite element prediction of the plastic Poisson’s ratio, *v*_*p*_, as a function of solids volume fraction, (ρ^*^/*ρ*_*s*_), from the unit cell calculations. The plastic Poisson’s ratio, *v*_*p*_, increased from 0.075 to 0.2 as (ρ^*^/*ρ*_*s*_) increased from 0.17 to 0.43.

Figure S2d shows finite element predictions of the nominal hardness, $\frac{H}{Y}$, versus *v*_*p*_ for an inverse opal material with 0.17 total solids volume fraction. The nominal hardness, $\frac{H}{Y}$, remained at approximately 1.3 when *v*_*p*_ was smaller than 0.3, and increased to 2.7 as *v*_*p*_ reached 0.5.

### Mechanical measurements

The inverse opal cellular solids were mechanically characterized using nanoindentation and micropillar compression. An Agilent G200 Nanoindenter with a diamond Berkovich tip measured the hardness and elastic modulus of the samples through continuous stiffness measurements. The continuous stiffness technique provided mechanical properties as a function of indentation depth. The indentation depths were limited to 7.5 μm of displacement or the maximum peak load of 600 mN. Figure S4 shows hardness measurements for various nickel cellular solids with 500 nm diameter pores coated with nickel and rhenium-nickel alloy. The data has a large standard deviation due to the surface roughness inherent to the porous structure at indentation depths below 500 nm. Material aggregation and substrate mechanical properties affect the measurements at indentation depths comparable to the sample thickness^64,65^. In between these extremes, the data has a plateau typically near 2000 nm depth. The hardness and elastic modulus from each indentation were determined by the average value in this plateau range. For each sample, the reported values were the average of 10 indentations or more.

Micropillar specimens were prepared by using an FEI Helios Nanolab 600i focused ion beam. Annular milling in three steps using beam currents of 21 nA, 2.5 nA and 80 pA resulted in cylindrical specimens of ≈3.6 μm diameter and ≈8 μm height. A final 100 nm-wide ring milling facilitated adjusting the height of the micropillars and minimizing their taper to less than two degrees from vertical. The Agilent G200 Nanoindenter with a 10 μm diameter flat diamond punch compressed the micropillars at an average strain rate of ≈1e-4 s^−1^. The nanoindenter deformed the micropillars to a total strain of about 20%. SEM-measured micropillar diameter, taken at the micropillar center, and height allowed the calculation of stress-strain behavior. The stress at 0.2% strain offset was used as the yield strength. The loading and unloading moduli were corrected to account for the substrate thickness using the method described in ref.^66^. The Poisson’s ratio of the inverse opal cellular solids was taken as zero for nanoindentation and micropillar compression calculations based on previous testing of nanoporous open-cell foams and qualitative experimental observations^3,8^. The micropillar unloading moduli were 2–5X greater than the loading moduli and were similar to moduli measured with nanoindentation.

## Supplementary information

Supplementary Info

## Data Availability

The datasets generated during and/or analyzed during the current study are available from the corresponding author on reasonable request.
