# Supplementary material for: High strength metallic wood from nanostructured nickel inverse opal materials
Source: Sci Rep. 2019 Jan 24;9:719. doi: 10.1038/s41598-018-36901-3 (PMC6345818; doi:10.1038/s41598-018-36901-3)
Supplement: Supplementary file 1 — Supplementary Info [file 41598_2018_36901_MOESM1_ESM.pdf]

Supplementary Materials for

High strength metallic wood from nanostructured nickel inverse opal materials

James H. Pikul, Sezer Özerinç, Burigede Liu, Runyu Zhang, Paul V. Braun, Vikram S. Deshpande, William P. King

correspondence to: pikul@seas.upenn.edu

This PDF file includes:

Supplementary methods
Supplementary references
Figs. S1 to S5

Supplementary methods

Volume fraction calculation

Figure S1a shows a unit cell of the inverse opal cellular solid and indicates important geometric parameters. The inverse opal volume is the cubic unit cell volume minus the volume of the sintered polystyrene spheres, V_{PS}. The conformally coated layer volume, V_{coat}, is the volume of a thin layer subtracted from V_{PS}. The symmetry of the FCC unit cell allows V_{coat} and V_{PS} to be calculated from the geometry of two neighboring PS spheres. Figure S1b shows the geometric model used to calculate the volume fractions. The circles with radius R represent two PS spheres in contact after opal self-assembly with an initial 0.74 volume fraction expected for FCC packing. The PS volume increases after sintering and is calculated by increasing R to R_e such that neighboring radii overlap by a length b. V_{PS} is the volume of 4 spheres of radii R_e minus the volume of 48 overlapping spherical caps of height h or

$$V_{PS} = 4\,\frac{4}{3}\,\pi\,{R_e}^3 - 48\,\frac{1}{3}\,\pi\,h^2(3\,R_e - h), \qquad \text{[S1]}$$

where $R_e = \sqrt{(b/2)^2 - R^2}$ and h is R_e - R. V_{coat} is the volume of the sphere of radius R_c subtracted from V_{PS}. Additionally, the volume where the coating does not deposit, marked with hatching, is integrated and subtracted out, but not including the volume of the spherical cap marked by h_2.

$$V_{coat} = V_{PS} - 4\,\frac{4}{3}\,\pi\,(R_c)^3 - 48\int_0^{\frac{R_c R}{R_e}} \pi\left(\frac{b}{2} - \sqrt{t^2 - x^2}\right)^2 dx + 48\frac{1}{3}\,\pi\,{h_2}^2(3\,R_c - h_2), \qquad \text{[S2]}$$

where h_2 is $R_c\,(1 - R\,/\,R_e)$ and t is $R_e - R_c$. $(\rho^*/\rho_s)_{Ni}$ and $(\rho^*/\rho_s)_{coat}$ are calculated from V_{PS} and V_{coat} normalized by the unit cell volume, $\left(2\,R\,\sqrt{2}\right)^3$.

$$\left(\frac{\rho^*}{\rho_s}\right)_{Ni} = 1 - \frac{V_{PS}}{\left(2\,R\,\sqrt{2}\right)^3} \ \text{and} \qquad \text{[S3]}$$

$$\left(\frac{\rho^*}{\rho_s}\right)_{coat} = \frac{V_{coat}}{\left(2\,R\,\sqrt{2}\right)^3}. \qquad \text{[S4]}$$

Equations S3 and S4 are used to generate a sample of volumes based on the exhausted combination of the measured R, b, and t. These representative samples determine the mean and standard deviations of ε_{Ni} and ε_{coat}. R and b are measured in SEM images of the inverse opal with no coating. R is half the average distance between the center of interconnect openings, that is, the distance from the center of b on one side of the PS void to the center of b on the other. b is the average measured interconnect diameter. t is measured by taking half the difference in the measured interconnect opening, b, before and after deposition. t is also measured directly at the inverse opal struts in cross-section SEMs, although this is difficult for coatings less than 15 nm.

The self-assembly process does not always result in a perfect FCC orientation of the PS spheres. Oversized PS particles cause the loss of contact between adjacent PS spheres, usually resulting in a small separation between spheres and rarely in a vacancy. As the number of defects increases the regular PS orientation can degenerate into a random close packed structure (RCP) resulting in a nickel volume fraction of 0.36 [1,2]. It is important to be able to quantify the level of order in inverse opal cellular solids and adjust the solids volume fraction accordingly. The coordination number of packed spheres has been shown to correlate to their volume fractions [1,3,4].

Figure S1c shows experimental data that relates coordination number to porosity, ν, combined with known FCC, BCC, and RCP coordination numbers [1,3,4]. A polynomial correlation of $\nu = 0.007\ CN^2 - 0.155\ CN + 1.1093$ was determined using the data in Fig. S1c, where ν is the void volume fraction of polystyrene spheres before sintering and CN is the average coordination number of the sample. CNs greater than 10 minimally affect the volume fraction. A FCC orientation has a CN of 12. When neighboring PS spheres are in contact and sintered, the resulting interconnect reduces the volume fraction of the inverted structure. When the CN is less than 12, the total volume lost from sintering is reduced and the starting volume fraction, ν, is increased. $(\rho^*/\rho_s)_{Ni}$ is corrected to $(\rho^*/\rho_s)'_{Ni}$ by subtracting the amount of volume lost due to sintering from ν, such that

$$\left(\frac{\rho^*}{\rho_s}\right)'_{Ni} = \nu - CN\frac{\left(\frac{\rho^*}{\rho_s}\right)_{Ni}(b) - \left(\frac{\rho^*}{\rho_s}\right)_{Ni}(b=0)}{12}, \quad \text{[S5]}$$

where $(\rho^*/\rho_s)_{Ni}(b)$ is the mean solved using Eq. 6 with exhausted combinations of R and b. When the inverse opal cellular solid is coated with additional material, the coating volume fraction, $(\rho^*/\rho_s)_{coat}$, is corrected to $(\rho^*/\rho_s)'_{coat}$ by multiplying the mean of $(\rho^*/\rho_s)_{coat}$ in Eq. 7 by 1/12 the CN and adding the volume fraction of a thin film that would cover the area where there are no interconnects between pores. The thin film volume fraction is 1/12 the volume fraction of a spherical shell made by the film thickness on the interior of a sphere with the diameter of the PS, multiplied by (12 – CN). The final total solid volume fraction is

$$\left(\frac{\rho^*}{\rho_s}\right)''_{Ni} = \left(\frac{\rho^*}{\rho_s}\right)'_{Ni} + \left(\frac{\rho^*}{\rho_s}\right)'_{coat}. \quad \text{[S6]}$$

In addition to defects in the ordered structure, cracks several micrometers wide separate ordered islands of PS particles. The cracks become fully dense nickel after nickel electrodeposition. These areas are avoided during inverse opal mechanical measurements.

Strut area calculation

We used measurements of the polystyrene radius, R, and interconnect diameter, b, to calculate the effective diameter of the inverse opal struts; the same measurements required to calculate the inverse opal volume fractions. Figures S1d and S1e show the geometry of the narrowest part of the strut, which has a pseudo-triangular shape with concave edges that result from the intersection of three spheres. The effective diameter, d, was calculated from the strut area, A, perimeter, P, and coating thickness, t, by

$$d = \frac{4A}{P} + 2t. \quad \text{[S7]}$$

t is measured experimentally as discussed in the volume fraction calculation section. The area of the strut is the area of the triangle that connects the strut tips with side length a minus the area of the 3 circular segments, Z.

$$Z = 0.5\left(R_e{}^2\varphi - 0.5a\sqrt{4R_e{}^2 - a^2}\right). \quad \text{[S8]}$$

The angle between strut edges from the pore center, φ, is $\pi/3 - 2\tan^{-1}(b/2R)$. The edge length between the strut tips, a, was calculated using the law of cosines with R_e and φ. R_e is $\sqrt{(b/2)^2 - R^2}$. The strut area is therefore

$$A = \frac{\sqrt{4}}{3}a^2 - 3\frac{1}{2}\left(R_e{}^2\varphi - \frac{1}{2}a\sqrt{4R_e{}^2 - a^2}\right). \quad [S9]$$

The strut perimeter is three times the arc length of the strut side,

$$P = 3s = 3R_e\varphi. \quad [S10]$$

Numerical simulations of the uniaxial compression test

The methods used to simulate the compression and the indentation tests of the inverse opal cellular solid are described here. The compression tests were simulated by explicitly modeling a unit cell of the cellular solid using three-dimensional (3D) finite element (FE) calculations. The calculations were carried out with periodic boundary conditions and the unit cell was described by an elastic-perfect plastic constitutive model with solid Nickel properties from experiments. The effective properties (Young's modulus, Yield strength, etc.) of the unit cell were then obtained from the calculations and compared with compression tests.

Since the cellular solid was subjected to a macroscopically homogeneous stress state during the uniaxial compression test, it was sufficient to consider a unit cell of the cellular solid to evaluate the effective mechanical properties, as illustrated in Fig. S2a. A periodic boundary condition (PBC) was enforced on the boundary $\partial\Omega$ of the unit cell Ω to ensure the strain-periodic displacement field. Here we give a brief description of the PBC, and the reader is referred to ref. [5] for a detailed discussion.

In general, for each pair of points (denoted as x^+ and x^-) on the boundary of the unit cell, we imposes the following relationship

$$u(x^+) - u(x^-) = \varepsilon^{IO}(x^+ - x^-); \forall x \in \partial\Omega, \quad [S11]$$

where u denotes the displacement field. ε^{IO} is the average strain tensor throughout the inverse opal unit cell,

$$\varepsilon^{IO} = \frac{1}{V_\Omega}\int_\Omega \varepsilon(x)dV, \quad [S12]$$

where $\varepsilon(x)$ is the strain field of the unit cell, and V_Ω is the total volume of the unit cell. To simulate the uniaxial compression test, we imposed a constant strain ε_{33}^{IO} in the 3 direction, and calculated the stress response σ_{33}^{IO}. The stress tensor was defined as

$$\sigma^{IO} = \frac{1}{V_\Omega}\int_\Omega \sigma(x)dV. \quad [S13]$$

The effective young's modulus E was then evaluated using

$$\sigma_{33}^{IO} = E\varepsilon_{33}^{IO}. \quad [S14]$$

The effective yield stress σ_Y was then calculated as the value of σ_{33}^{IO} at 0.2 offset strain.

The simulation was carried out using the implicit version of the commercial finite element package ABAQUS[6], with ten-node nonlinear tetrahedron elements. The periodic boundary

condition was implemented using Python scripts[7]. The unit cell was modeled with isotropic elasticity and J2 plasticity, using the solid Ni property measured in experiments.

Indentation mechanism of a cellular solid

Indentation of a cellular solid by a self-similar indenter such as Berkovich tip proceeds in a self-similar manner. Fig. S3 illustrates this concept with a contour of the plastic strain at selected normalized indentation depths, h/L. During indentation, material densifies around the indenter (i.e. deforms and attains its densification strain, $\hat{\varepsilon}_P \approx 0.5$) and no further deformation takes place in that region. Further deformation is concentrated in a small zone immediately in the vicinity of the deformed region, which deforms until that region densifies and indentation proceeds by then inducing deformation further into the material. This self-similar deformation mechanism implies that the material properties (modulus and strength) extracted from indentation are independent of the densification process as the indentation is governed by deformation of the cellular solid prior to densification. This mechanism has been extensively reported in the literature; see for example ref. [8-11]. The caveat is that for this continuum mechanism to be applicable, the indented zone needs to be much larger than the cell size of the cellular solid. This mechanism is clearly seen in Fig. S4 where the measured hardness first decreases with increasing indentation depth and then plateaus out after the indenter tip continues past several inverse opal unit cells, confirming the self-similar nature of the indentation process.

Discrepancy between measurements and predictions

The nanoindentation calculations underpredicted hardness at higher solid volume fractions, see Fig. 4a. The measured H/Y ratios approached 3 at high volume fractions, which suggests that the plastic Poisson's ratio of the inverse opals, v_p, approached 0.5. In our calculations, we used lower values of v_p as our unit cell simulations (Fig. S2c) predicted that $v_p < 0.2$ for all solid volume fractions investigated here. We believe that the effective values of v_p were higher in the experiments compared to calculations because imperfections in the micro-structure allowed early contact between cell walls, which resulted in the inverse opals behaving in a more incompressible manner. These imperfections are not accounted for in the idealized unit cell simulations.

Supplementary references

1 van de Lagemaat, J., Benkstein, K. D. & Frank, A. J. Relation between Particle Coordination Number and Porosity in Nanoparticle Films: Implications to Dye-Sensitized Solar Cells. *The Journal of Physical Chemistry B* **105**, 12433-12436, doi:10.1021/jp013369z (2001).

2 Mason, G. A model of the pore space in a random packing of equal spheres. *Journal of Colloid and Interface Science* **35**, 279-287, doi:http://dx.doi.org/10.1016/0021-9797(71)90121-4 (1971).

3 Benkstein, K. D., Kopidakis, N., van de Lagemaat, J. & Frank, A. J. Influence of the Percolation Network Geometry on Electron Transport in Dye-Sensitized Titanium Dioxide Solar Cells. *The Journal of Physical Chemistry B* **107**, 7759-7767, doi:10.1021/jp022681l (2003).

4 Ni, M., Leung, M. K. H., Leung, D. Y. C. & Sumathy, K. An analytical study of the porosity effect on dye-sensitized solar cell performance. *Solar Energy Materials and Solar Cells* **90**, 1331-1344, doi:http://dx.doi.org/10.1016/j.solmat.2005.08.006 (2006).

5 Michel, J.-C., Moulinec, H. & Suquet, P. Effective properties of composite materials with periodic microstructure: a computational approach. *Computer methods in applied mechanics and engineering* **172**, 109-143 (1999).

6 Systèmes, D. ABAQUS Theory Guide, v6. 13. *Dassault Systèmes, Waltham, MA* (2013).

7 VanRossum, G. & Drake, F. L. *The python language reference*. (Python software foundation Amsterdam, Netherlands, 2010).

8 Hodge, A. M. *et al.* Scaling equation for yield strength of nanoporous open-cell foams. *Acta Materialia* **55**, 1343-1349, doi:http://dx.doi.org/10.1016/j.actamat.2006.09.038 (2007).

9 Onck, P., Andrews, E. & Gibson, L. Size effects in ductile cellular solids. Part I: modeling. *International Journal of Mechanical Sciences* **43**, 681-699 (2001).

10 Khaderi, S. N. *et al.* The indentation response of Nickel nano double gyroid lattices. *Extreme Mechanics Letters* **10**, 15-23, doi:https://doi.org/10.1016/j.eml.2016.08.006 (2017).

11 Liu, R. & Antoniou, A. A relationship between the geometrical structure of a nanoporous metal foam and its modulus. *Acta Materialia* **61**, 2390-2402 (2013).

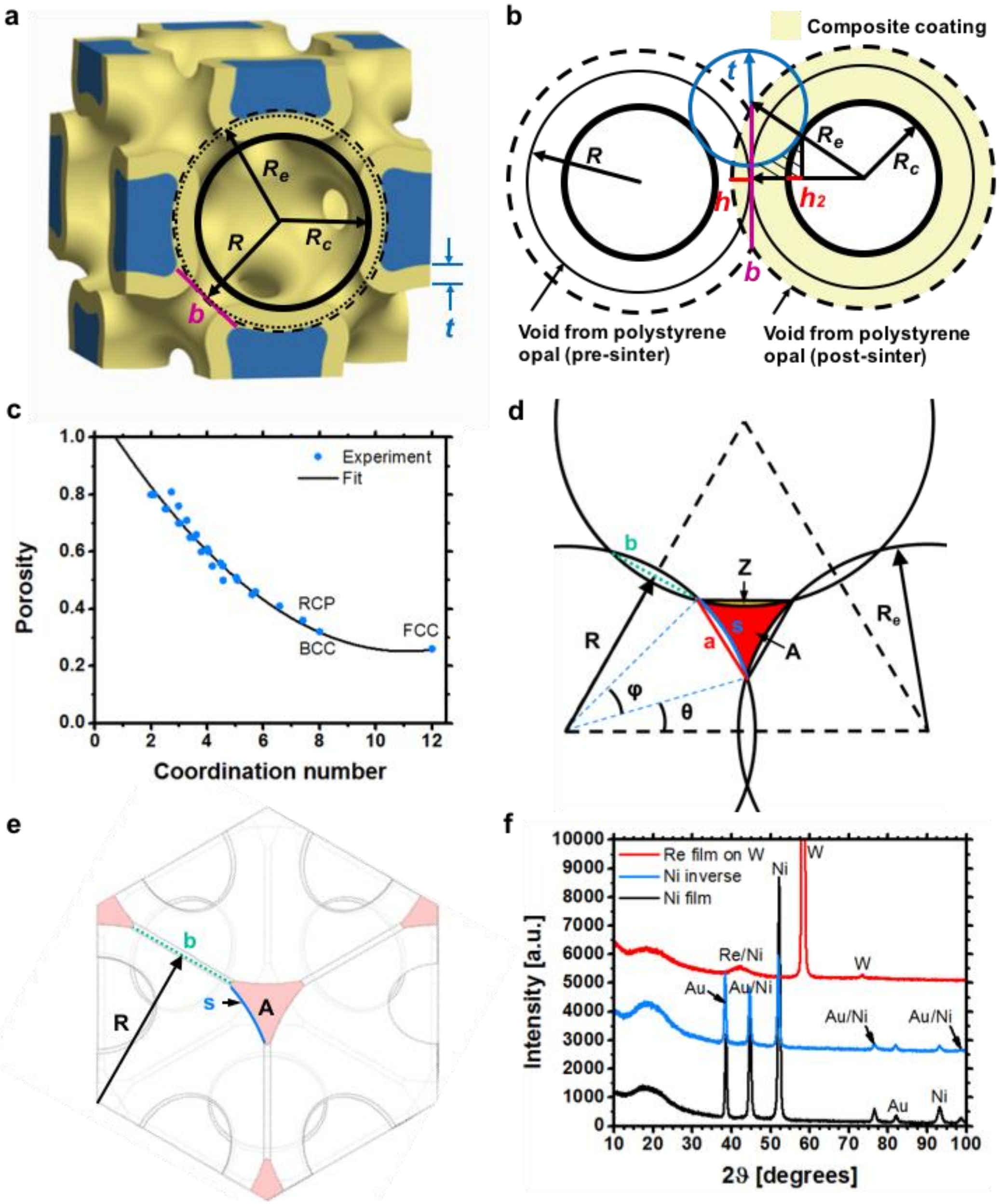

Figure S1 a) A unit cell of the inverse opal material, showing key geometric parameters. b) A geometric model of the inverse opal material used to calculate volume fraction. c) Data and curve fit relating the porosity of packed spheres to the coordination number. Experimental data is from [1,3,4]. The curve fit is used to correct the volume fraction of the inverse opal material for defects during assembly. d) A diagram of the strut geometry related to the polystyrene particle geometry after self-assembly and sintering. The effective strut diameter is calculated from measurements of *R* and *b*. e) A 2-D wireframe of the inverse opal unit cell top. f) X-ray diffraction data for an electrodeposited nickel film, nickel inverse opal material, and rhenium film used to characterize the crystallinity of the composite materials.

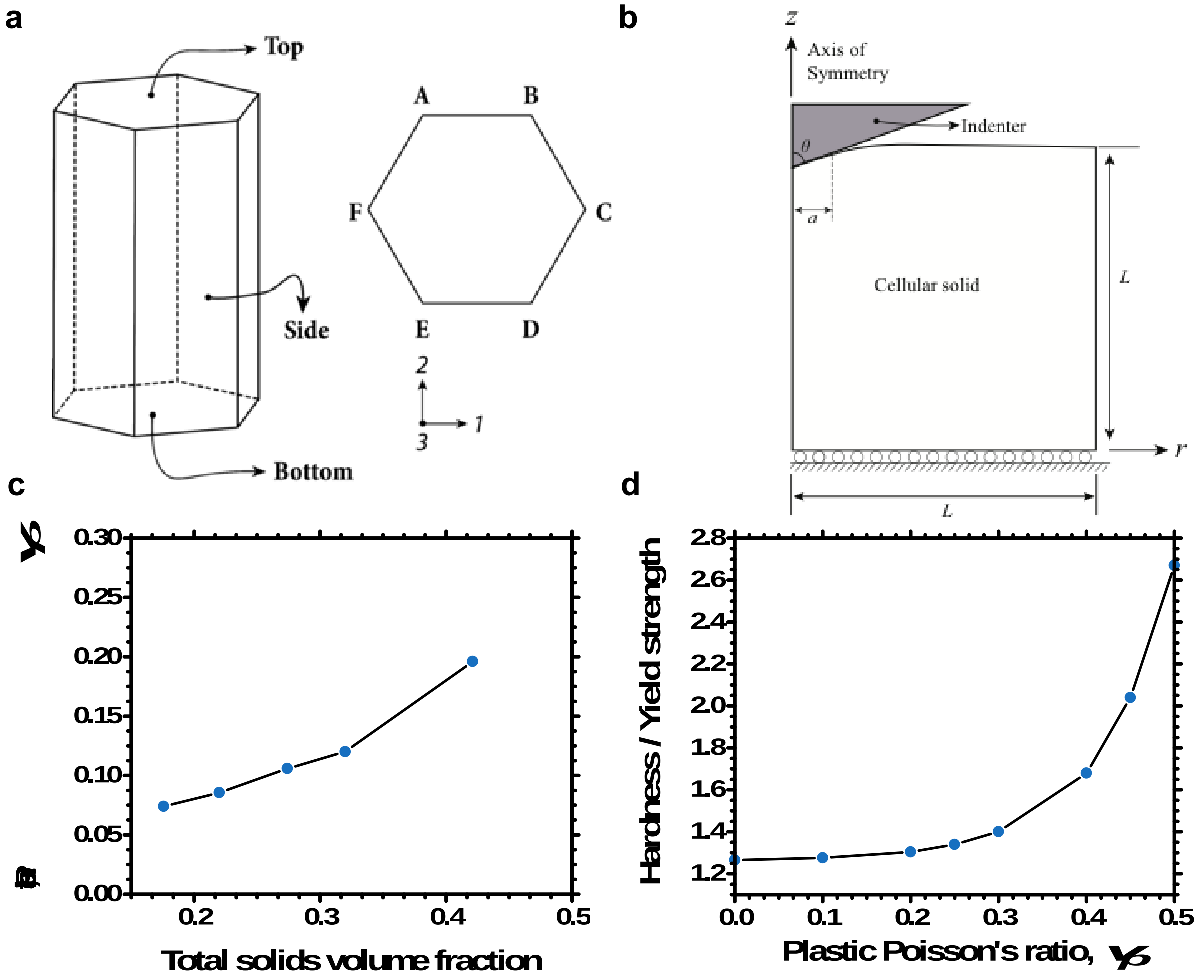

Figure S2 a) A diagram of the hexagonal unit cell used in the simulations. b) A diagram of the indentation boundary value problem using cylindrical coordinates. A conical indenter is forced into a block of cellular solid at the center. c) Plastic Poisson's ratio as a function of relative density. d) Hardness divided by flow stress at 10% strain as a function of plastic Poisson's ratio for an inverse opal material with 0.17 solids volume fraction.

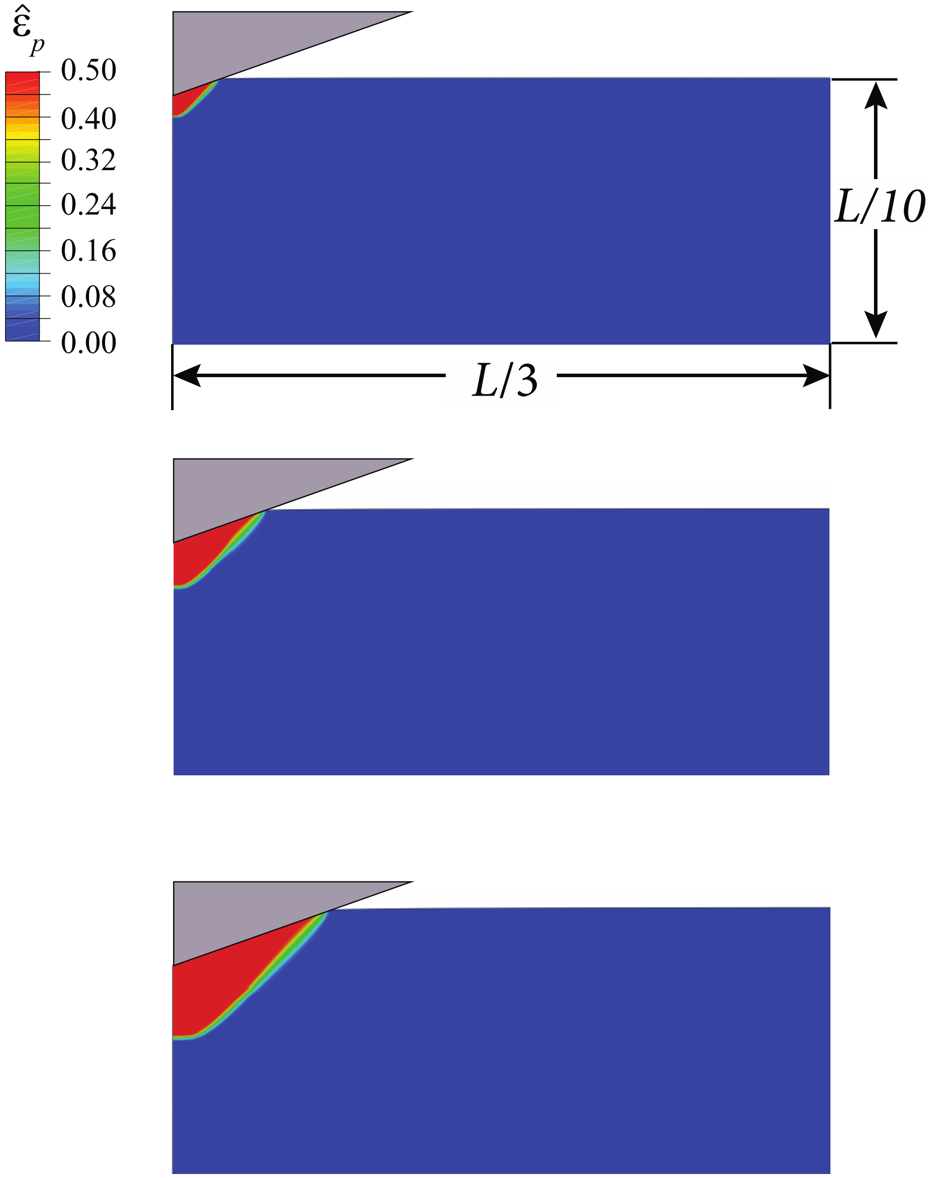

Figure S3 Finite element simulations of the effective plastic strain ε̂_P immediately under the conical indenter at three selected values of normalized indentation depth h/l=0.01,0.02 and 0.03.

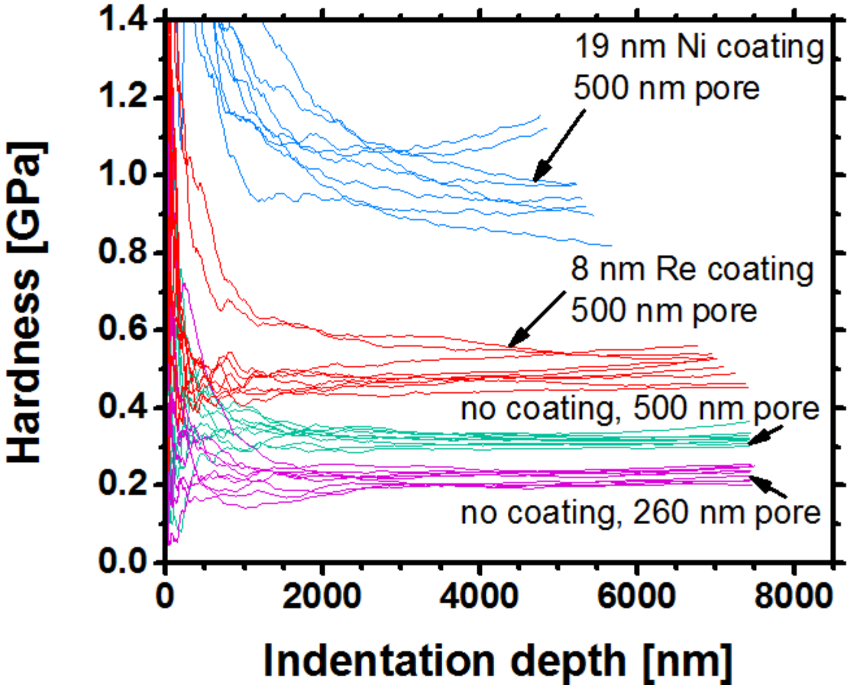

Figure S4 Nanoindentation hardness measurements of the inverse opal materials.

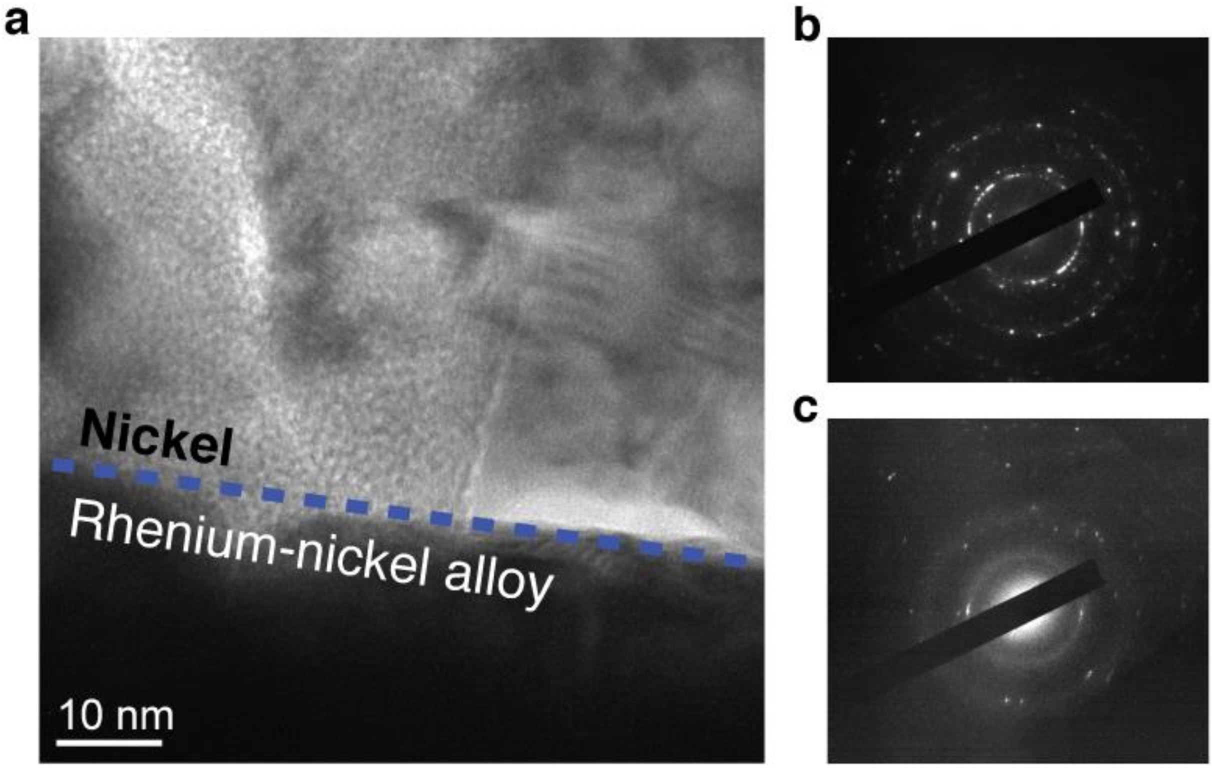

Figure S5 a) Transmission electron micrograph of a nickel inverse opal coated with rhenium-nickel alloy. The blue dashed line shows the approximate separation between the two materials. b) TEM diffraction pattern of the nickel inverse opal. c) TEM diffraction pattern of the rhenium-nickel alloy.
